# The Differential Effects of a Selective Kappa-Opioid Receptor Agonist, U50488, in Guinea Pig Heart Tissues

**DOI:** 10.1155/2015/906039

**Published:** 2015-03-01

**Authors:** Chi-Feng Hung, Hsin-Ju Li, Hsun-Hao Chang, Gon-Ann Lee, Ming Jai Su

**Affiliations:** ^1^School of Medicine, Fu Jen Catholic University, No. 510 Zhongzheng Road, Xinzhuang District, New Taipei City 24205, Taiwan; ^2^Big Data Research Centre, Fu Jen Catholic University, New Taipei City, Taiwan; ^3^Department of Chemistry, Fu Jen Catholic University, New Taipei City, Taiwan; ^4^Department of Cardiology, Tainan Municipal Hospital, No. 670 Chongde Road, East District, Tainan 70173, Taiwan; ^5^Department of Pharmacology, College of Medicine, National Taiwan University, No. 1 Jen-Ai Road, Section 1, Zhongzheng District, Taipei 10051, Taiwan

## Abstract

The differential effects of a selective kappa- (*κ*-) opioid receptor agonist, U50488, were elucidated by monitoring the contraction of isolated guinea pig atrial and ventricular muscles. In electrically driven left atria, U50488 in nanomolar concentration range decreased the contractile force. Norbinaltorphimine (norBNI), a selective *κ*-receptor antagonist, and pertussis toxin (PTX) abolished the negative inotropic effect of U50488. In contrast, the inhibitory effect was not affected by the pretreatment of atropine or propranolol. Even though U50488 exerted a negative inotropic effect in the left atrium, it did not affect the contractile force of the right atrium and ventricles paced at 2 Hz. Similarly, the beating rate of the spontaneously beating right atrium was also unaffected by U50488. These results indicate that the activation of *κ*-opioid receptors can only produce negative inotropic effect in left atria via activation of PTX-sensitive G protein in guinea pigs. The absence of negative inotropic effects in right atria and ventricles suggests that there may be a greater distribution of functional *κ*-opioid receptors in guinea pig left atria than in right atria and ventricles, and the distribution of the receptors may be species-specific.

## 1. Introduction

Many studies have indicated that *κ*-opioid receptors exist in the heart by receptor binding assay [1–3] and physiological studies [4, 5]. Stimulation of *κ*-opioid receptors in the heart may evoke negative inotropic [6, 7] and chronotropic effects [8]. Prior investigators also suggested that activation of cardiac *κ*-opioid receptors could mediate cardioprotective and antiarrhythmic effect during myocardial ischemia and reperfusion [9–12]; modulation of cardiac function by opioid peptide receptor agonists or antagonists, and future drug development to improve myocardial salvage would be possible [2, 3].

While U50488, a selective *κ*-opioid receptor agonist, decreases the electrically induced [Ca^2+^]_i_ transient in rat cardiac myocytes at a higher concentration (*μ*mol/L) by activating the phosphoinositol pathway [13, 14], it inhibits the augmentation of the electrically induced [Ca^2+^]_i_ transient by *β*-adrenoceptor stimulation in the heart at a lower concentration (nmol/L) [13]. Electrophysiological studies showed that U50488 could inhibit the P-type calcium channel in brain Purkinje cells through activation of *κ*-opioid receptors [15, 16]; it could also inhibit the L-type calcium, sodium, and potassium channels in ventricular myocytes directly at a higher concentration [17, 18].

Several studies have reported that 5-hydroxytryptamine (5-HT), calcitonin gene-related peptide (CGRP), angiotensin II, somatostatin, adenosine, and diadenosine tetraphosphate changed the contractile force more in atrial than in ventricular tissues [19–23]. However, opioid receptors exist in both atria and ventricles; it is still unknown whether there is any response difference in the *κ*-opioid receptors between these tissues. This study was planned to examine the effects of a selective *κ*-receptor agonist, U50488, on atrial and ventricular muscles isolated from guinea pigs.

## 2. Materials and Methods

### 2.1. Chemicals

NorBNI (norbinaltorphimine), U50488 (trans-3,4-dichloro-N-methyl-N-[2-(1-pyrrolidinyl)cyclohexyl]-benzeneacetamide), U69593 ((+)-(5*α*,7*α*,8*β*)-N-methyl-N-[7-(1-pyrrolidinyl)-1-oxaspiro[4.5]dec-8-yl]-benzeneacetamide), and PTX (pertussis toxin) were purchased from Research Biochemicals International (RBI) Chem. Co. (USA). Propranolol, atropine, and carbachol were purchased from Sigma Chem. Co. (USA).

### 2.2. Isolated Cardiac Preparations and Mechanical Response

Preparations of isolated left and right atria (or ventricles) from male guinea pigs (Hartley strain, 0.2–0.5 kg) were used. The preparations were bathed in Tyrode solution, and the Tyrode solution (composition in mM: NaCl 137.0, KCl 5.4, MgCl_2_ 1.1, NaHCO_3_ 11.9, NaH_2_PO_4_ 0.33, dextrose 11.0, and CaCl_2_ 1.8) was aerated with 5% CO_2_ and 95% O_2_ at 37 ± 0.5°C. Contractions of spontaneously beating right atrial preparations, as well as the electrically driven left atrial, right atrial, and right ventricular strips, were measured by connecting one end of the preparation to a force displacement transducer (type BG 25, Gould Inc., Cleveland, Ohio, USA) by a fine silk thread and were recorded on a Gould RS 3400 recorder. To obtain the maximum developed tension, an optimal preload (1.0 g) was used. Left atria, right atria, and right ventricular strips were stimulated at a frequency of 2 Hz by rectangular pulse of 2 ms duration at supramaximal intensity via an isolated Grass S88 stimulator (Grass Instruments Co., Quincy, MA, USA).

### 2.3. Assessment of the Effects of PTX

To assess a possible role of pertussis toxin-sensitive G protein in the actions of U50488, guinea pigs were pretreated with PTX 150 *μ*g/kg (i.p.) for 24 h before sacrifice. The influence of PTX on cardiac tissues was verified by the contractile responses of left atrial strips to carbachol. Carbachol (0.1 *μ*mol/L) decreased the basal contractile force from 0.8 ± 0.1 g to 0.5 ± 0.1 g (decrease about 44% in control group, *n* = 5), whereas a less decrease was found, from 0.8 ± 0.1 g to 0.6 ± 0.1 g (decrease about 24%, *n* = 5), in PTX-treated group.

### 2.4. Statistics

Data are expressed as mean ± SE. Statistics comparison was made by Student's paired or unpaired *t*-test with the level of significance taken as *P* < 0.05.

## 3. Results

### 3.1. The Differential Effects of *κ* Receptor Agonists on Cardiac Contractility

**Figure 1:**
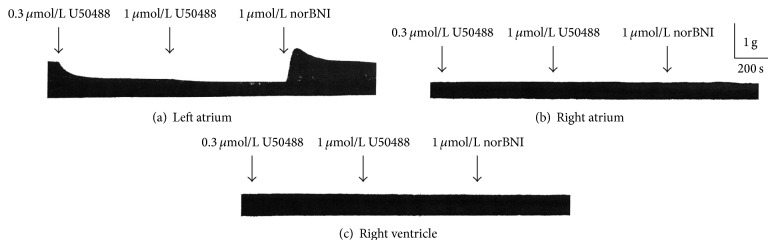
Effect of U50488 on the contractile force in guinea pig atria and ventricle. Sinoatrial node of right atrium was removed. Atria and ventricle were paced at 2 Hz. After equilibrium for 30 min, U50488 and norBNI were added sequentially. (a) Left atrium. (b) Right atrium. (c) Right ventricle.

**Figure 2:**
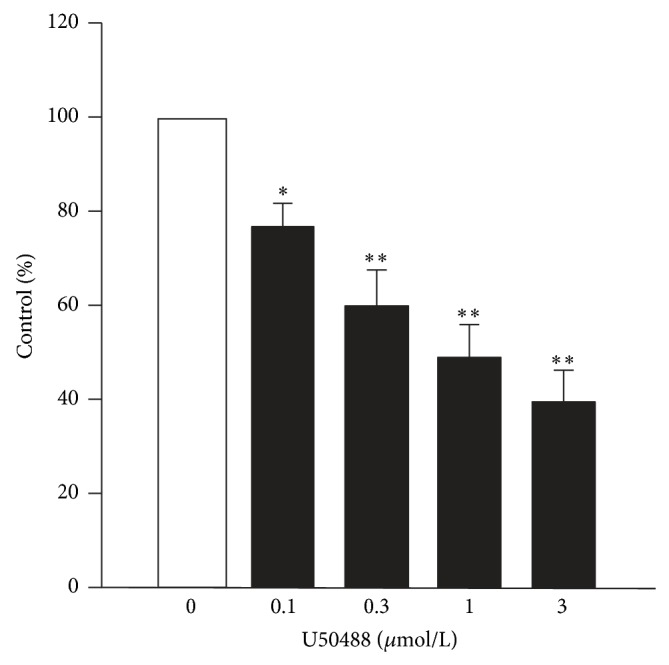
The concentration-dependent effects of U50488 on contractile force of left atrium illustrated as percentage of control. Vertical lines are SE. ∗ and ∗∗ indicate *P* < 0.05 and *P* < 0.01 as compared with control (*n* = 5).

Figure 1 shows the original tracings of continuous tension recordings of three different guinea pig atrial and ventricular preparations in cumulative response to 300 nmol/L and 1 *μ*mol/L U50488. In the left atrium, U50488 exerted a significant and immediate negative inotropic effect (Figure 1(a)). This inhibitory effect reached a steady state in 10 min and could be eliminated by norBNI (1 *μ*mol/L), a selective *κ*-opioid receptor antagonist. In contrast, the inhibitory effect was not found in the preparations of right atrium, right ventricle (Figures 1(b) and 1(c)), and left ventricle (not shown). Average data of the influence of U50488 on the contractile force of left atrial preparations are shown in Figure 2. U50488 started to reduce the contractile force significantly at 30 nmol/L. At 100 nmol/L, 300 nmol/L, and 1 *μ*mol/L, it reduced the force to 77.3 ± 5.0%, 60.6 ± 7.7%, and 49.8 ± 6.9% (*n* = 5) of the control, respectively. Similarly, we found that U69593, another selective *κ*-receptor agonist, also has the same negative inotropic effect on left atria but not the preparations of right atria and ventricles (figure not shown) in our other experiments. The inhibitory effect could also be abolished by norBNI (1 *μ*mol/L). At 300 nmol/L and 1 *μ*mol/L, it reduced the force to 54.4 ± 5.3% and 45.9 ± 6.4% (*n* = 4) of the control, respectively. As higher concentrations of U50488 and U69593 may elicit effects not mediated via opioid receptor [24], they were not investigated with the *κ*-opioid receptor antagonist.

### 3.2. Effects of norBNI and PTX on the Negative Inotropic Action of U50488

**Figure 3:**
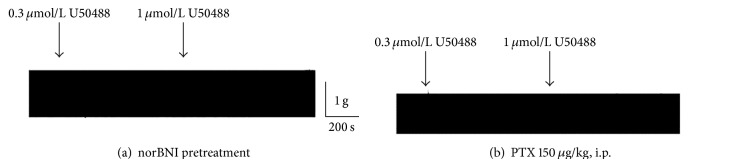
Representative tracings show the effect of norBNI and PTX on the effect of U50488 in left atria. (a) A norBNI (1 *μ*mol/L) pretreated atrium. (b) An atrium from PTX-pretreated guinea pig (150 *μ*g/kg for 24 h).

**Figure 4:**
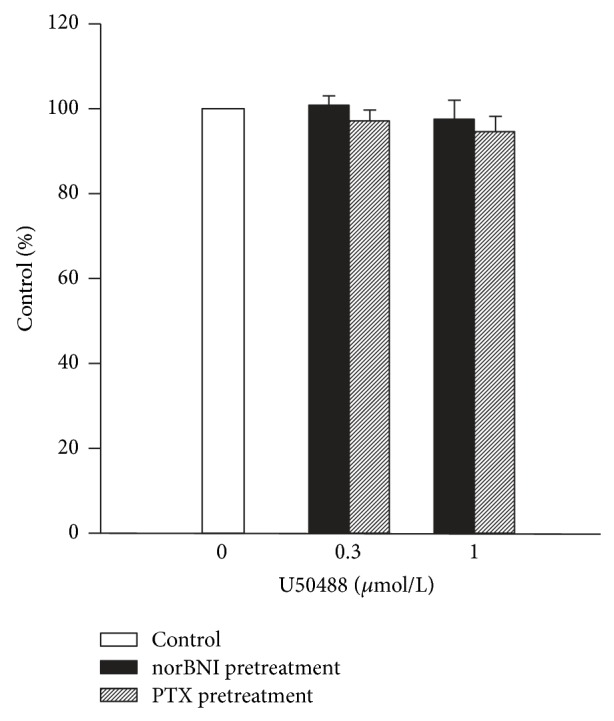
Effect of U50488 on left atria pretreated with norBNI (*n* = 4) and PTX (*n* = 5).

To further evaluate whether the inhibitory effects of U50488 on left atria were due to *κ*-opioid receptors and the coupling of inhibitory G protein, G_i/o_, the muscle strips were pretreated with a selective *κ*_2_-receptor antagonist, norBNI (1 *μ*mol/L), for 10 min. We found that norBNI at 1 *μ*M alone did not affect the contractile force of the left atrial preparations, but it prevented the inhibitory effects of U50488 (Figure 3(a)). In left atria isolated from guinea pigs pretreated with PTX (150 *μ*g/kg, i.p.), which catalyzes the adenine nucleotide ribosylation of G_i/o_ protein *α*-subunits, U50488 also failed to exert any inhibitory response (Figure 3(b)). The average data of U50488 after norBNI or PTX pretreatment are shown in Figure 4.

### 3.3. Effects of Propranolol and Atropine on the Negative Inotropic Action of U50488

**Figure 5:**
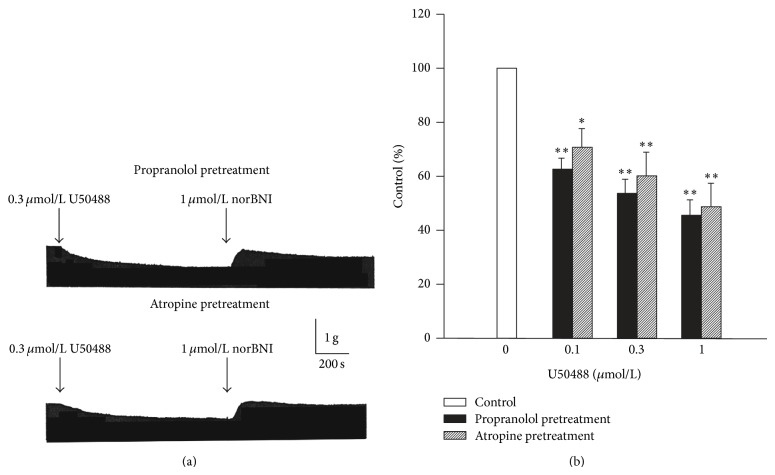
Negative inotropic effect of U50488 in left atria pretreated with propranolol (3 *μ*mol/L, *n* = 6) or atropine (1 *μ*mol/L, *n* = 5). (a) Representative traces show the effect of U50488 on the contractile force in left atria pretreated with propranolol or atropine for 20 min. (b) Concentration-dependent effects of U50488 on contractile of left atria in the presence of propranolol or atropine. Values are expressed as percentage of control. Vertical lines are SE. ∗ and ∗∗ indicate *P* < 0.05 and *P* < 0.01 as compared with control.

It had been reported that propranolol could modify the inhibitory effect of U50488 in isolated right atria of rat [4, 7], and the *κ*-receptor agonist could inhibit norepinephrine release from cardiac sympathetic nerve [25]. Therefore, we evaluated whether the inhibitory effects of U50488 in isolated left atria of guinea pig could be affected by propranolol (3 *μ*mol/L). A representative example of the effect of propranolol pretreatment on the U50488-induced negative inotropic action is shown in Figure 5(a), and the average result is shown in Figure 5(b). The average amplitudes of the contractile force of the left atrial preparations were decreased to 62.7 ± 4.3%, 54.2 ± 5.1%, and 46.0 ± 5.7% (*n* = 6) of the control by 0.3, 1, and 3 *μ*mol/L of U50488, respectively. Decreases in contractile force induced by 0.3 and 1 *μ*mol/L of U50488 were also completely eliminated by norBNI. In comparison with the decrease in contractile force induced by U50488 alone, propranolol did not affect the negative inotropic effect of U50488 on left atrial preparations. Considering the release of acetylcholine being another possibility which may contribute to the negative inotropic effect of U50488, we examined the negative inotropic effect of U50488 on left atria pretreated with atropine (1 *μ*mol/L). Our data showed that the negative inotropic effect of U50488 was unaffected by atropine. The average amplitudes in contractile force were decreased to 70.6 ± 7.0%, 60.4 ± 8.6%, and 49.0 ± 8.6% (*n* = 5) of the control by 0.3, 1, and 3 *μ*mol/L of U50488, respectively (Figures 5(a) and 5(b)).

### 3.4. Effect of U50488 on the Spontaneous Beating Rate of Guinea Pig Right Atria

**Figure 6:**
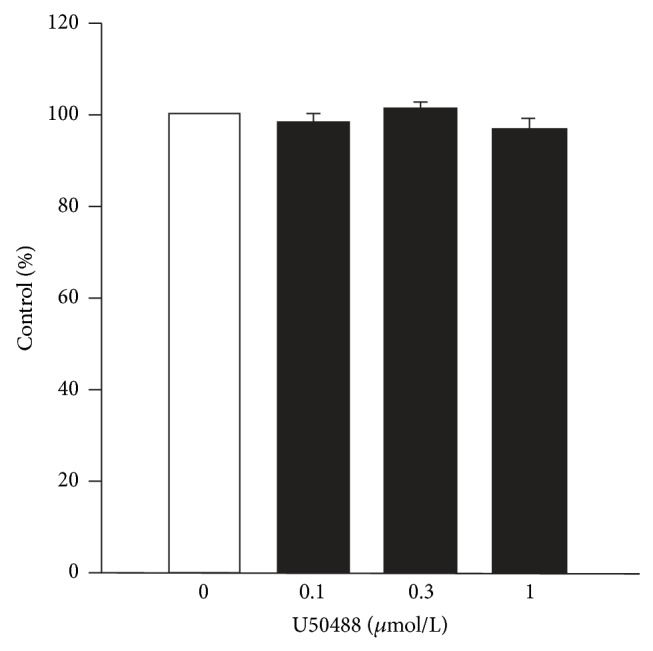
Effect of U50488 on spontaneously beating rate of right atria in guinea pig. Values are presented as percentages of control (*n* = 6).

U50488 could evoke negative chronotropic effect on isolated right atria of rat [6, 7]. However, we did not find any change in the spontaneous beating rate in guinea pig right atria in our study (Figure 6).

## 4. Discussion

In this study, we compared the inotropic effects of U50488 and U69593, two selective *κ*-opioid receptor agonists, in guinea pig left atria to their effects in right atria and ventricles. We observed that both U50488 and U69593 dose-dependently decreased the contractile force of guinea pig atrial muscles, and this effect was exerted only in left atria. The absence of negative inotropic effect in right atria and ventricles suggests that *κ*-opioid receptors in these two regions may be less or absent in guinea pigs. However, missing signaling pathway for coupling of *κ*-opioid receptor activation to negative inotropism in these two cardiac tissues may also be another possibility. These speculations remain for further identification, and radioligand binding studies using appropriate radioligands may be necessary.

Corresponding to previous observations which showed the coupling of the *κ*-opioid receptor to activation of the G_i/o_ protein [16], our results showed that the negative inotropic effect of U50488 on left atrial tissues could be abolished by norBNI and PTX, which suggests that the action is mediated through *κ*-opioid receptors by activation of PTX-sensitive G protein. At a higher concentration (3 *μ*mol/L), U50488 was found to decrease the contractile force of right ventricular preparations that could not be eliminated by norBNI in this study. The negative inotropic effect at the higher concentration of U50488 may be due to its directly inhibitory effect instead of the activation of *κ*-opioid receptors. These observations agree with the study that showed a direct inhibition of calcium current in guinea pig ventricular myocytes by U50488 at similar concentration range [24]. The 15.0 ± 4.0% (*n* = 6) inhibition of the contractile force of right ventricular strips by 3 *μ*M U50488 in our study is comparable to the 18% inhibition of calcium current in guinea pig ventricular cell by Utz et al. [24].

Previously, U50488 had been shown to exert negative inotropic effects in both atrial and ventricular preparations by the activation of *κ* receptors in rats [7]. The different results in this study may be due to the difference in experimental conditions or animal model. In addition, Niroomand et al. [26] have shown that functional *κ*-opioid receptors may not present in canine cardiac sarcolemma because the *κ*-receptor agonist, U50488, did not inhibit adenylate cyclase activities. These observations strongly suggest that the distribution of *κ*-opioid receptors in cardiac myocardium may be species-specific. Therefore, functional study of the *κ*-opioid receptor on human cardiac preparation is necessary.

In general, agents and interventions that increase K^+^ conductance (G_k_) shorten action potential duration (APD) and tend to have negative inotropic effects, whereas agents that decrease K^+^ current lengthen APD and may have positive inotropic effects [27]. Therefore, the shortening of the action potential duration is a possible mechanism responsible for the negative inotropic effect of U50488. In our unpublished data, we found that U50488 could shorten the duration of action potential and decrease the contractile force in guinea pig left atrial preparations. This effect could also be eliminated by the selective *κ*-opioid receptor antagonist, norBNI (1 *μ*mol/L). Because the delayed rectifier and inward rectifier potassium channels play a key role during repolarization of action potential in guinea pig [28–30], the shortening of action potential may be related to these channels being affected by U50488. However, further voltage clamp studies will be needed to characterize the effect of U50488 on the delayed rectifier or inward rectifier potassium current.

To our knowledge this report demonstrates for the first time that the opioid receptor agonist elicits a negative inotropic response in left atria without having a corresponding effect in right atria and ventricles. Whether the different effects of U50488 exist in human tissues remains to be studied. Several studies have shown that postischemic contractile dysfunction or “stunning of myocardium” occurred especially during ischemia-reperfusion [31–33]. During a period of stunning, left ventricular ejection fraction may be significantly impaired. Myocardial stunning may delay recovery from cardiogenic shock or left ventricular failure. Therefore, it is important to avoid stunning of ventricular tissue during ischemia-reperfusion. While there is an increase in *κ*-opioid peptide release in myocardial ischemia-reperfusion [34], the absence of significant negative inotropic properties of *κ*-opioid receptor agonist in the right ventricle may be beneficial to cardiac performance during ischemia-reperfusion. In addition, decrease in contractile force in the left atrium may slightly alleviate the loading of the left ventricle and be beneficial to the failing left ventricular myocardium.
